# The Reflux Disease Questionnaire: a measure for assessment of treatment response in clinical trials

**DOI:** 10.1186/1477-7525-6-31

**Published:** 2008-04-30

**Authors:** Michael Shaw, John Dent, Timothy Beebe, Ola Junghard, Ingela Wiklund, Tore Lind, Folke Johnsson

**Affiliations:** 1 Park Nicollet Clinic and University of Minnesota Medical School, Minneapolis, MN 55416-2699, USA; 2 Department of Gastroenterology and Hepatology, Royal Adelaide Hospital, Adelaide, SA 5000, Australia; 3 Mayo Clinic College of Medicine, Rochester, MN 55905, USA; 4 AstraZeneca R&D, SE-431 83 Mölndal, Sweden; 5 University of Lund, SE-221 00 Lund, Sweden

## Abstract

**Background:**

Critical needs for treatment trials in gastroesophageal reflux disease (GERD) include assessing response to treatment, evaluating symptom severity, and translation of symptom questionnaires into multiple languages. We evaluated the previously validated Reflux Disease Questionnaire (RDQ) for internal consistency, reliability, responsiveness to change during treatment and the concordance between RDQ and specialty physician assessment of symptom severity, after translation into Swedish and Norwegian.

**Methods:**

Performance of the RDQ after translation into Swedish and Norwegian was evaluated in 439 patients with presumed GERD in a randomized, double-blind trial of active treatment with a proton pump inhibitor.

**Results:**

The responsiveness was excellent across three RDQ indicators. Mean change scores in patients on active treatment were large, also reflected in effect sizes that ranged from a low of 1.05 (dyspepsia) to a high of 2.05 (heartburn) and standardized response means 0.99 (dyspepsia) and 1.52 (heartburn). A good positive correlation between physician severity ratings and RDQ scale scores was seen. The internal consistency reliability using alpha coefficients of the scales, regardless of language, ranged from 0.67 to 0.89.

**Conclusion:**

The results provide strong evidence that the RDQ is amenable to translation and represents a viable instrument for assessing response to treatment, and symptom severity.

## Background

Symptom-focused questionnaires have an important role in clinical trials of gastroesophageal reflux disease (GERD) management. This is especially the case given that symptom relief is a major goal of treatment for patients with GERD [1], and that patient self-report on symptom status is now believed to be more reliable than physician assessment [2]. Critical needs for symptom evaluation in clinical trials include optimizing symptom-based selection of research subjects for the trial, evaluating baseline symptom severity, and assessing response to treatment. These aims need to be achievable with brief, easily scored questionnaires that are preferably self-administered. The multicenter, multinational nature of pharmaceutical clinical trials also requires questionnaires that are amenable to translation into multiple languages.

The Reflux Disease Questionnaire (RDQ), a 12-item self-administered questionnaire, was designed to assess the frequency and severity of heartburn, regurgitation, and dyspeptic complaints and to facilitate the diagnosis of GERD in primary care [3]. The psychometric properties of the RDQ have been examined in a primary care population. Internal consistency reliability levels were high, with alpha coefficients ranging from 0.80 for the dyspepsia scale to 0.81 and 0.85 for the heartburn and regurgitation scales, respectively. In terms of stability, the test-retest reliability coefficients ranged from 0.80 to 0.88. An assessment of change scores among a subset of patients provided initial evidence of the responsiveness of the RDQ regurgitation and heartburn scales to treatment effects. Based on these preliminary results, the RDQ may have the potential to meet some of the questionnaire needs for GERD clinical trials.

In this study, the performance of the RDQ was assessed in a clinical treatment trial for patients with GERD. Whereas prior work on the RDQ was completed with patients seen in primary care, the current investigation was undertaken in the context of a multicenter, double-blind, randomized study in which Scandinavian patients with heartburn as the predominant symptom were treated with esomeprazole for 2 weeks. We extended the earlier psychometric work on the RDQ by investigating its responsiveness to changed symptom status as a result of therapy in a large clinical population of patients diagnosed as having GERD. The concordance between the RDQ evaluations of symptom severity was compared to those offered by specialty physicians. The success of the translation of the RDQ into Swedish and Norwegian was also evaluated.

## Methods

### Patients

Adult patients presenting with presumed GERD symptoms were recruited from 35 endoscopy units across Sweden and Norway [4,5]. Inclusion criteria specified that the main symptom should be heartburn of six months duration or longer. Also, patients were required to have had heartburn episodes on four days or more during the seven days prior to the one on which they were enrolled. Exclusion criteria included irritable bowel syndrome (IBS) or any current or historical evidence of a primary esophageal motility disorder other than reflux disease, as judged by an investigator. Additional exclusion criteria were major complications of GERD (such as esophageal stricture, ulcer and/or Barrett's metaplasia and/or significant dysplastic change in the esophagus), the presence of active gastric or duodenal ulcer or erosive duodenitis, or esophagitis grade C or D according to the Los Angeles classification system [6] at the initial screening endoscopy. Eligible patients were randomly assigned in double-blind fashion to two weeks of therapy in one of three arms: 1) esomeprazole 20 mg twice daily (n = 176); 2) esomeprazole 40 mg once daily (n = 171); or 3) placebo (n = 92), in the proportions 2:2:1. For the purpose of this evaluation the two active treatment groups were pooled, as the results in the two groups were essentially the same.

### GERD diagnosis

All patients underwent an endoscopy and pH monitoring, including assessment of Symptom Association Probability (SAP). A diagnosis of GERD was considered 'proven' when either endoscopy (LA grade A or B) and/or pH monitoring (> 3.4% of the total time or > 3.2% of the supine time with intragastric pH < 4) or SAP (95% or more during the 24 hr pH monitoring) was positive.

### Measures

#### The Reflux Disease Questionnaire (RDQ)

The RDQ is a self-administered questionnaire in which subjects are asked to report the frequency and severity of their upper gastrointestinal symptoms. There are three subscales that evaluate regurgitation, heartburn, and dyspepsia [3]. The heartburn and regurgitation subscales can be combined into a GERD dimension. In the published survey, the time referent is symptoms that have occurred over the last four weeks. In this study, the time referent was the last four weeks at baseline, but one week at the post-treatment visit (visit 2, after two weeks of treatment). Item content includes the following: 1) four items on the frequency and severity of acid taste in the mouth and movement of materials upwards from the stomach (Regurgitation scale); 2) four items measuring the frequency and severity of pain or burning behind the breastbone (Heartburn scale); and 3) four items on the frequency and severity of pain or burning in the upper stomach (Dyspepsia scale). Response options were scaled as Likert-type with scores ranging from 0 to 5 for frequency (not present to daily) and severity (not present to severe). Each subject's score was calculated as the mean of item responses with higher scores indicating more severe or frequent symptoms. The psychometric properties of the RDQ are described in more detail by Shaw and colleagues [3].

#### Overall Treatment Evaluation (OTE)

The OTE, a validated scale, rates the change in symptoms on a 15-point scale (-7 to -1 = worse; 0 = no change; and +1 to +7 = better) [7-9]. At the second clinic visit, patients were asked to fill in the OTE questionnaire and rate if their symptoms were better, worse, or unchanged. If their symptoms had changed, patients were asked to rate the magnitude of improvement or worsening on a seven-point scale ranging from 1 to 7. In the present analysis worsening was collapsed into one category, a little better was defined as +1 to +4, while much better was defined as +5 to +7.

#### Other assessments

At both clinic visits, a clinical trial assessment interview and a physical examination were conducted by the investigators. Patients were asked about the severity of their heartburn, regurgitation, dysphagia, epigastric pain, and nausea over the three days prior to each clinic visit, this inquiry being structured by the trial case record form for each visit and graded 0 = none, 1 = mild, 2 = moderate, and 3 = severe.

### Translation and cultural adaptation

The RDQ was translated into Norwegian and Swedish according to international principles [10]. The translators met with members of the RDQ survey team to maintain content and clarity of the questionnaire. As part of the translation process, the Swedish and Norwegian language versions were tested with GERD patients. The RDQ was back translated into English after translation into both languages and reviewed again by members of the RDQ survey team to ensure preservation of content and clarity of the items.

### Analytical Strategy

There were three specific analytical objectives: 1) assessment of the responsiveness to treatment of selected RDQ scales; 2) assessment of the concordance between RDQ- and physician-generated ratings of disease severity; and 3) assessment of the internal consistency reliability of the translated versions to verify the consistency of the concepts. Responsiveness was determined by comparison of RDQ data to global symptom change reported on the OTE question. The change from baseline should be larger for patients who were 'better' according to OTE than for those who were 'worse or 'unchanged'. Student's t test for paired samples, the standardized response mean [11], and the effect size [12] were also calculated. The associations between the RDQ dimensions heartburn, regurgitation and dyspepsia, and the corresponding symptom severity assessments made by the physician is a measure of the ability of RDQ to measure what it is intended to measure. Physician severity assessment was compared to RDQ data with Pearson correlation coefficients and one-way analysis of variance (ANOVA) on mean scale score differences across physician severity rating categories. Internal consistency refers to the extent to which the items within a scale are interrelated. High values would imply that the items within a scale belong together also after the translation. The consistency of the translation across three languages was estimated by calculating the internal consistency reliability using Cronbach's alpha [13].

## Results

### Patients

134 subjects from Norway and 305 from Sweden with a mean age of 51.4 (13.5) years were enrolled at 35 sites. The baseline characteristics and clinical information for patients with data from both a baseline and subsequent visit are provided in Table 1. GERD was proven in 82% of subjects while in 18% the diagnosis was based solely on symptoms. Symptom severity as judged by investigators and the RDQ and the response to esomeprazole treatment was not different for those with proven GERD as opposed to those in whom objective testing was negative.

**Table 1:** Demographic and clinical characteristics at initial visit

	**n = 439**	**%**
**Gender**		
Male	224	51
**Age**		
< 50	188	43
50–64	166	38
> 65	85	19
**Country**		
Norway	134	31
Sweden	305	69
**Endoscopic grading**		
Normal	199	45
Grade A	135	31
Grade B	105	24
**Method of GERD diagnosis**^a^		
Endoscopy	240	55
pH monitoring	270	62
Symptom Association	95	22

**Total proven diagnoses**	**354**	**81**

^a ^Percentages do not add up to 100 due to multiple methods of diagnosis.

### Responsiveness

After two weeks of trial therapy, patients were told to assess symptoms during the previous seven days and completed the RDQ a second time. Responsiveness was first examined by collapsing responses on the OTE question to four possibilities (worse, the same, a little better, and much better), as described above. A progressive increase was seen in the change score moving from the worse to much better categories, regardless of the treatment group (Table 2). Table 3 shows effect sizes and standardized response means.

**Table 2:** Change in the RDQ scales from baseline to 2 weeks, by patient rating of the Overall Treatment Evaluation (OTE)

**OTE**	**N**	**Heartburn**		**Regurgitation**		**Dyspepsia**		**GERD**^a^	
		
		**Mean**	**SD**	**Mean**	**SD**	**Mean**	**SD**	**Mean**	**SD**
**Esomeprazole**									
Worse	6	0.04	2.00	-0.42	0.41	-0.08	1.47	-0.19	1.20
The same	36	0.76	1.48	0.68	1.11	0.31	1.24	0.71	0.99
A little better	43	1.60	1.46	1.52	1.08	1.27	1.64	1.56	0.97
Much better	231	2.74	1.26	2.25	1.42	1.94	1.59	2.51	1.08
All	316	2.31	1.52	1.92	1.47	1.64	1.66	2.12	1.26
**Placebo**									
Worse	3	0.42	1.18	-0.25	0.25	0.50	0.43	0.08	0.63
The same	26	0.42	1.54	0.37	1.30	0.27	1.41	0.37	1.08
A little better	28	0.88	1.0	1.05	1.10	0.78	1.20	0.90	0.72
Much better	22	2.10	1.26	1.38	1.82	1.65	1.68	1.74	1.33
All	79	1.05	1.43	0.86	1.43	0.86	1.50	0.93	1.17

^a^Heartburn and regurgitation subscales combined. SD, standard deviation.

**Table 3:** Responsiveness indicators for all patients by esomeprazole and placebo treatment

**RDQ Scale**	**Effect size**		**Standardized response mean**	
	
	**Esomeprazole**	**Placebo**	**Esomeprazole**	**Placebo**
Heartburn	2.05	0.85	1.52	0.74
Regurgitation	1.36	0.63	1.31	0.60
Dyspepsia	1.05	0.56	0.99	0.57
GERD^a^	2.13	0.93	1.68	0.79

^a^Heartburn and regurgitation subscales combined.

The observed effect sizes ranged from a low of 1.05 (dyspepsia) to a high of 2.05 (heartburn). As anticipated, the responsiveness of the heartburn scale was highest and the dyspepsia scale the lowest of the three scales.

It must be noted that a sizable change in scores was observed for those in the placebo group who indicated they were at least a good deal better on the OTE item.

**Figure 1:**
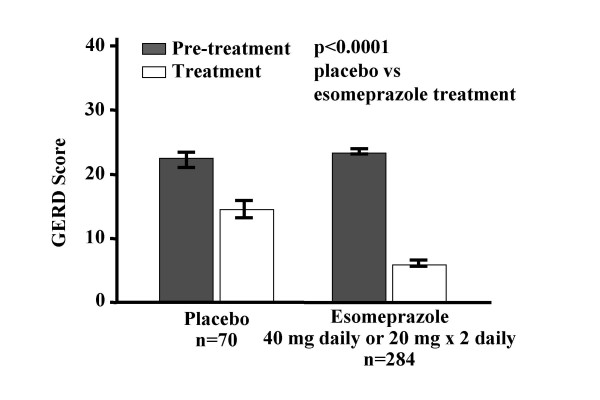
Responsiveness of GERD Score.

Esomeprazole in either dose was markedly more effective than placebo in improving the GERD score (p < 0.0001) (Figure 1). Esomeprazole in either dose improved the GERD score (mean change 2.12; 95% confidence interval: 1.98, 2.26) markedly more effectively than placebo (mean change 0.93; 95% confidence interval: 0.91, 1.43), p < 0.0001.

### RDQ and Physician Severity Rating Concordance

Table 4 depicts the relationships between the scores on the three RDQ scales and physician symptom severity ratings for regurgitation, heartburn, and dyspepsia at baseline and at visit 2. A positive correlation was found between physician severity ratings and RDQ scale scores, which increased with the investigator ratings of symptom severity. The observed correlations were strongest at the follow-up visit (see bolded coefficients).

**Table 4:** Correlations between RDQ scale scores and clinical severity assessments at baseline and visit 2

**RDQ Scale**	**Clinical Severity Assessment**			
	
	**Regurgitation**	**Heartburn**	**Dyspepsia**	**GERD**
**Baseline**				
Regurgitation	**0.54**	0.14	0.09	0.51
Heartburn	0.21	**0.33**	0.20	0.29
Dyspepsia	0.26	0.17	**0.47**	0.18
GERD^a^	0.51	0.29	0.18	-
**Visit 2**				
Regurgitation	**0.63**	0.56	0.40	0.62
Heartburn	0.49	**0.74**	0.42	0.73
Dyspepsia	0.53	0.67	**0.58**	0.46
GERD^a^	0.62	0.73	0.46	-

^a^Heartburn and regurgitation subscales combined.

Values are Pearson's correlation coefficients. Results in bold denote those within similar domains.

### Internal consistency of the translated RDQ

High levels of internal consistency across the translated RDQ scales would be evidence of the amenability to translation. Analysis revealed that, regardless of language, all but one of the alpha coefficients for the scales of heartburn, regurgitation, dyspepsia and GERD (Norway: 0.67, 0.8, 0.88, and 0.72, respectively; Sweden: 0.75, 0.86, 0.89 and 0.78, respectively) surpassed the accepted level of 0.70 [14].

## Discussion

The RDQ was developed to facilitate the identification of GERD in primary care and this was the setting in which its psychometric properties were established [3]. This study demonstrated the utility of the RDQ to evaluate treatment response in a clinical trial of a new medication. The questionnaire effectively differentiated various levels of patient-assessed symptom severity compared to physician-assessed severity. Consistency of performance in two languages was also observed. The study population, being highly enriched for GERD, precluded determination of the predictive validity of the RDQ for a GERD diagnosis.

The responsiveness of the RDQ scales to treatment was observed to be quite high by all three methods of analysis. The observed effect sizes outstripped conventional thresholds for superior responsiveness [15]. While, as anticipated, the responsiveness was somewhat lower for the dyspepsia scale, it too was quite large. These results provide clear evidence that the RDQ is sensitive to clinically important change in the context of a treatment trial. The effect sizes noted in the placebo group, as a whole, were clearly lower than those in the active treatment group. When split according to the OTE responses, which measure the patient's perception of improvement, the effect sizes were more or less comparable to those in the active treatment group. However, only 22 patients reported that they were 'much better' in the placebo group compared to 231 in the esomeprazole-treated group, indicating the superiority of active therapy.

An important aspect of a useful symptom questionnaire is its ability to capture nuances in various disease symptom complexes. Evidence that an instrument is able to capture severity would be particularly useful because disease severity often directs different courses of treatment. The purpose of the current study was to evaluate how well the RDQ tracked physician ratings of disease severity for regurgitation, heartburn, and dyspepsia. The results demonstrated that the RDQ is quite sensitive to symptom severity as measured by specialty physicians. The fact that the concordance between the two sources was more pronounced at the follow-up visit may be due to several factors, e.g. practice effects (on both the patient's and physician's part), compression of symptom evaluations at the follow-up visit in response to treatment (most patients got better), and/or a more comparable time referent between both data sources at the follow-up. The results of a recent paper argue for the use of a self-report survey to supplement investigator obtained data in this critical step, especially if the primary outcome measurement tool is going to be a self-report symptom measure [16]. In this study, before treatment, the concordance between how physicians and patients rated symptom severity of heartburn and epigastric pain was only modest and moreover, the physicians consistently underestimated symptom severity. If complete symptom resolution was achieved there was good agreement after treatment between physician and patient ratings; with increasing severity of remaining symptoms, the concordance decreased significantly.

Development of subjective, self-report questionnaires for symptom assessment requires rigorous psychometric evaluation. Development and psychometric evaluation of instruments includes item selection and multitrait scaling, internal consistency of items that combine into a dimension [17], as well as confirmation of convergent and/or discriminant validity. Although such validation is a necessary component of instrument development, it is not sufficient to guarantee that the instrument will perform well when used in an actual clinical trial setting where responsiveness to change is the most important criterion. Coupled with the prior work on the RDQ [3], the results of the current investigation provide strong evidence that the RDQ represents a viable instrument for assessing symptom severity, subject selection and response to treatment in clinical trials of GERD. Work focusing on the performance of the RDQ for epidemiological survey (or tool) and for GERD diagnosis in primary care is currently underway.

## Conclusion

This study provides evidence that the RDQ is amenable to translation into Norwegian and Swedish, and that it represents a viable instrument for assessing symptom severity and response to treatment in clinical trials of patients with GERD.

## Competing interests

The authors declare that they have no competing interests.

Ola Junghard and Tore Lind are AstraZeneca employees, and Ingela Wiklund was an employee at the time of the study.

## Authors' contributions

FJ was the principal investigator in the study, and was involved in the design. MS, TB, TL, OJ, and JD were involved in the analysis, reporting and writing up of the manuscript. All authors read and approved the final manuscript.
